# Membranes Coated with Graphene-Based Materials: A Review

**DOI:** 10.3390/membranes13020127

**Published:** 2023-01-19

**Authors:** Despina A. Gkika, Vasiliki Karmali, Dimitra A. Lambropoulou, Athanasios C. Mitropoulos, George Z. Kyzas

**Affiliations:** 1 Department of Chemistry, International Hellenic University, 65404 Kavala, Greece; 2 School of Mineral Resources Engineering, Technical University of Crete, 73100 Chania, Greece; 3 Department of Chemistry, Aristotle University of Thessaloniki, 54124 Thessaloniki, Greece

**Keywords:** membranes, graphene-based membranes, performance, separation

## Abstract

Graphene is a popular material with outstanding properties due to its single layer. Graphene and its oxide have been put to the test as nano-sized building components for separation membranes with distinctive structures and adjustable physicochemical attributes. Graphene-based membranes have exhibited excellent water and gas purification abilities, which have garnered the spotlight over the past decade. This work aims to examine the most recent science and engineering cutting-edge advances of graphene-based membranes in regard to design, production and use. Additional effort will be directed towards the breakthroughs in synthesizing graphene and its composites to create various forms of membranes, such as nanoporous layers, laminates and graphene-based compounds. Their efficiency in separating and decontaminating water via different techniques such as cross-linking, layer by layer and coating will also be explored. This review intends to offer comprehensive, up-to-date information that will be useful to scientists of multiple disciplines interested in graphene-based membranes.

## 1. Introduction

Over the last twenty years, separation via membranes has been deemed as an innovative technology suitable to address massive challenges, such as lack of resources and the environmental impact of human activities [1]. Contrary to typical techniques, membrane separation has become popular due to its exemplary advantages [2] as a low-energy and eco-friendly option that takes up less space and can be performed continuously [3].

A membrane is essentially a selective boundary with pores or channels. They are more cost-efficient and effective, and their use is fundamental in eco-friendly technologies [4]. An ideal candidate offers high selectivity and flux and is more stable after appropriately tuning the pore size and shape. Furthermore, making the membrane less thick allows it to become more permeable and improves its efficiency [1]. Even though the potential of various membranes has already been demonstrated ever since early in the 20th century, their large-scale use did not become widespread for many decades [4]. Polymeric membranes are a very large part of the worldwide market; however, they have certain drawbacks such as the Robeson performance limit (permeability versus selectivity balance), the need to be de-fouled after use and their susceptibility to high temperatures and extreme environments, which prompted the request for alternative membrane materials [5]. As a result, the demand for new materials exhibiting high selectivity, an improved separation efficacy and better fouling resistance have increasingly risen [6].

Graphene and graphene oxide (GO) have demonstrated marked potential in separation processes using membranes, owing to being accessible and because of their strength and notable resistance to various types of solvents [7]. The inclusion of graphene in a polymer as a strengthening agent has enhanced the general efficiency and attributes of such compounds, which has been supported by the majority of researchers on the field [8].

In this context, additional effort has been devoted to comprehending their behavior and exploring the uses of materials based on graphene [9]. Graphene and its derivatives are emerging at a global level as components for the construction of highly performing innovative membranes, owing to their honeycomb-like structure and mass transport ability [9,10]. Furthermore, they are highly permeable and are great in molecular sieve applications, solvent-resistant, thermally and chemically stable, and mechanically strong [10,11]. The unique architecture and surface make them highly permeable and with almost no transportation resistance [10]. Specifically, sp^2^ hybridization and thicker atomic layers expedite the formulation of ultrathin selective barriers with expected separation capacity [10]. The introduction of selective defects allows for water molecule permeability through a pristine graphene monolayer, which is typically impermeable to gases [10,12]. The separation relies on the sieving ability of nanochannels in the membrane according to molecular size. Sub-nanometer pores offer little flow resistance and achieve extremely fast water permeance, while leger molecules are prevented from passing through [13]. It should be noted, however, that the introduction of defects results in leakages that are the actual barrier to the larger scale use of graphene-based membranes. Thus, the susceptibility to leakage is yet one more challenge to address [10,13].

The pristine graphene layer was found to be impenetrable to liquids and gases because of the very small gaps in its structure [5]. Graphene is renowned for its remarkable properties, such as electron mobility [14], great thermal conductivity, which is comparable to diamond and Carbon Nanotubes (CNTs) and almost a dozen times higher than copper [15], with a strength of approximately 42 N/m (more than 300 times that of steel) [16]. Similarly to graphene, GO has been featured in relevant literature because of its singular features that might improve the anti-fouling capacity by boosting the permeability and selectivity [17].

Considering the issues discussed, graphene-based membranes produced through diverse processes possess specific structures and transport pathways, allowing their application in multiple membrane processes such as water desalination [18,19,20], removal of dyes [21,22], and oil–water or gases separation [23,24].Since receiving awards for “ground-breaking experiments regarding the two-dimensional material graphene” in 2010, researchers have focused on graphene-based separation membranes [2,6,25,26,27,28,29], thus verifying that graphene and its derivatives are essential in these processes and demonstrating the maturity of the field, which requires a thorough overview to examine the latest advancements, trends and available opportunities.

This work delves into the most recent (past four years) research findings on the topic of GO-based membranes, going over the vast and scattered literature to obtain and organize the information on the synthesis approaches, the potential challenges related to them and the recorded data on the separation and decontamination efficiency. The novelty of this article is its mission to review the very recent literature and curate up-to-date (and easily comparable) information on the synthesis methods of graphene-based membranes, their applications and efficiency in water purification, bringing attention to the factors affecting the separation/decontamination processes. Appropriate information regarding the pollutants susceptible to each process is further collected and assessed, along with details on the rejection rates achieved.

## 2. Graphene Derivatives

Graphene quantum dots (GQD) is a term used for single or few-layer graphene nanosheets, often during studies of the photoluminescence property. GQDs have generally extremely small size, usually less than ten nm (and an average of five nm), which places them at the lower end of the graphene nanosheets’ range, whose upper end starts at less than 100 nm [30].

The reduction of graphene oxide can be conducted via chemical, thermal, electrochemical and photochemical approaches, resulting in the production of graphene-like materials, such as reduced graphene oxide (rGO) and highly reduced GO (HRG), etc. In spite of the considerable differences between pristine graphene and HRG, they still possess remarkable similarities. Their physicochemical attributes define this step as one of the most significant processes of GO, especially for bulk applications. The attributes of HRG produced via GO-reduction, are highly dependent on the reduction processes [31].

Turbostratic carbon is a 3D sp2-bonded carbon material, with layers whose carbon atoms don’t have a spatial relationship with the atoms in other nearby layers [30].

Graphene-based materials, which are also mentioned as graphene or graphene-family nanomaterials—refer to a group of 2D materials containing the name ‘‘graphene’’, multilayered materials (less than ten layers) included. They may be chemically modified forms (graphene oxide, reduced graphene oxide), or materials produced using graphene, its oxide, or other graphene material as a component [30].

Multi-layer graphene (MLG)—a two dimensional material in sheet form, comprising of a limited number (ranging between two and ten) of precisely-defined, stacked layers [30]. They may be found as either free flakes or substrate-bound coating,

Few-layer graphene (FLG)—a subgroup of MLG with an average of two to five layers. Examples include graphite nanoplates, nanosheets and nanoflakes; They are two dimensional with ABA or ABCA stacking, whose thickness does not exceed 100 nm [30].

Exfoliated graphite—a multilayer version, produced through partial graphite exfoliation (thermal, chemical, or mechanical), creating thin multilayer products that maintain the three-dimensional structure of graphite [30].

Graphene nanosheet—a single layer of hexagonally placed, sp2-bonded carbon atoms that is not the main part of a carbon material, but is either free or adhered on an external substrate, whose lateral dimension does not exceed 100 nm [30,32].

Metal oxides: In order to further improve and expand the applications of graphene, a wide range of metal and metal oxide nanoparticles have been combined with graphene. The CVD method requires the dissolution of carbon in metal surfaces, such as nickel and copper that serve as catalysts and then by cooling the metal to forcing it to separate. Metal and metal oxide nanocompounds are produced by chemically reducing metal precursors such as chloroauric acid, silver nitrate and potassium tetrachloroplatinate, using hydrazine hydrate, amines, and sodium borohydride. The combined effect of graphene and the metal-based materials in these nanocompounds have a new research pathway, including biomedical applications [31].

Polymer Nanocomposites: The binding or loading of metal and metal oxide nanoparticles on graphene for the preparation of graphene-based nanocomposites is commonly achieved in two different ways: post immobilization or in-situ binding. Post immobilization requires combining different graphene nanosheets options and pre-produced nanoparticles. Prior to that, the nanoparticles and/or the surface of graphene sheets are functionalized to improve the ability to process the end results, which greatly improves their solubility and thus offers additional alternatives for the creation of graphene-based compounds. Nevertheless, post immobilization might be affected negatively if the structure of the graphene sheets is not dense enough or if the coverage by the graphene sheets is irregular [31].

## 3. Summary of Graphene’s Physicochemical Properties and Synthesis Approaches

Before discussing the actual membranes, the inherent attributes and the creation process of graphene-based components will be summarized, because they heavily impact the preparation process and general efficiency.

### 3.1. Physicochemical Properties

There is a plethora of articles describing the simple yet perfect structure of graphene, with its atoms placed in a honeycomb-looking lattice [9], that led to the discovery of various unexpected mechanical, electronic and thermal properties, gaining global interest. The majority of the reviewed studies have not used the pristine form because of its limited outcomes. Materials based on graphene, however, such as GO, rGO and functionalized reduced graphene oxide (frGO), have become more popular, since they exhibit attributes similar to graphene [33,34,35].

The functional groups that can be found on the surface of component based on graphene offers the potential of formulating bonds with proteins, whereas the structure of pristine graphene allows for hydrophobic π–π interaction to lead any hydrophobic leftovers to the graphene surface [36]. Furthermore, its attributes could be adjusted to produce rGO and GO [26]. The difference between graphene and its oxide lies in the presence of functional groups that can easily affect the physicochemical properties of the GO and its derivatives. The hydrophilic groups facilitate the dissolution of graphene oxide sheets in wet environment, thereby easily processing and stacking sheets. Additionally, they offer reactive sites for GO modifications, impacting the architecture and attributes of the produced composite membranes [1].

Li et al. demonstrated that graphene is a very effective additive [37]. Using a surfactant may enhance the hydrophilic behavior and stability of the resulting composites. Leenaerts et al. highlighted graphene’s hydrophobicity [38]. The bond among water and graphene is not as strong as the one among water molecules. This behavior verifies the hydrophobic nature of graphene, while the presence of a nanostructure on its surface makes it more hydrophobic. As a result, water molecules may easily go through graphene sheets without getting attached, raising the flux. Furthermore, foulants cannot easily attach to the surface, thus hindering the fouling of the membrane [39].

### 3.2. Synthesis Processes

**Figure 1:**
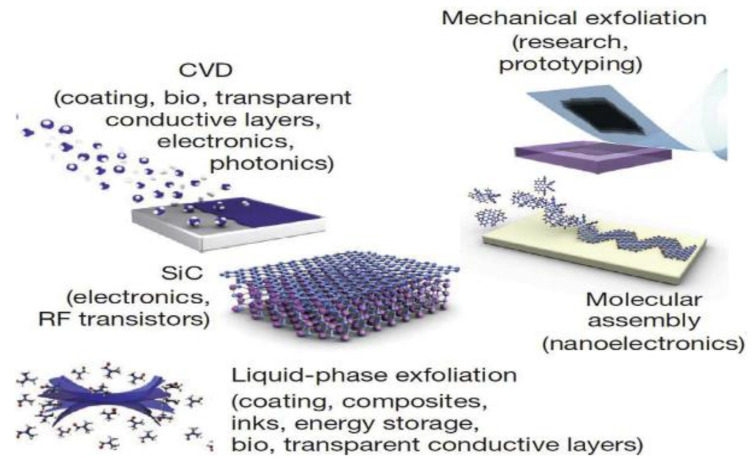
The five graphene and graphene derivative production mechanisms according to specific requirements and intended applications. Reprinted with permission from Ref. [8].

There are two main synthetic pathways, top-down and bottom-up. Graphene can be synthesized via various approaches (Figure 1), such as (1) manual exfoliation from bulk graphite or automatically via sonication; (2) liquid phase reduction of GO (3) film growth; (4) chemical vapor deposition of single layers; and (5) reduction of its derivatives. There are more than 1090 research works on the chemical vapor deposition (CVD) approach over the past decade, rendering it the most explored of the afore mentioned options [40].

Mechanical exfoliation was the first successful method of producing graphene by consecutive peelings via scotch tape [41]. Separated layers can later be placed on various substrates. Despite being simple and inexpensive, exfoliation is restrictive in regards to controlling the dimensions and quality of the layers [42]. Consequently, chemical exfoliation was deemed as an adequate starting point for future approaches with fewer limitations. Synthesis methods for MXene and graphene are similar. Graphene and MXene can be produced by the exfoliating graphite and MAX materials respectively. Modifying the properties of both graphene and MXene for some applications follows similar patterns [43]. Graphene and its synthesis were first discussed by Katsnelson et al. [44] who stated that the benzene-ring structure of closely connected carbon atoms of materials such as graphite and nanotubes was called graphene. The attributes and behavior of graphene depends on the number of its layers; thus, the capacity to be produced in films with varied thickness dictate its practical applications. For instance, the metallic behavior of the two-layer version is lower than that of the single layer [45]. Garaj et al. [46], employed an ion-implantation synthesis approach. The optical microscopy measurement yielded 2 μm nickel films, whose implantation was conducted using the Varian semiconductor equipment. The resulting surface atomic thickness corresponds to half, one, two and three graphene single layers. With higher carbon content, the graphene thickness increases, whereas the defective content is reduced [45].

The growth of thin graphene films has proven to be a successful approach for the synthesis of graphene [47]. Xu et al. presented the creation of very thin films within twenty minutes [48]. The growth on a crystal copper surface, and the effortless fusion of graphene enables the extremely swift formulation of films that are highly crystalline. This was accomplished via the fabrication of a single copper crystal that was annealed in gradient temperatures under a constant oxygen supply.

CVD is an effective approach for mass manufacturing of graphene-based membranes. The technique’s advantage is the ability to continuously prepare graphene films using an ever growing list of potential substrates, such as copper, platinum and nickel [49]. The chemical properties of graphene do not facilitate the relocation of graphene from one substrate to another, which might also result in imperfections and wrinkles in the membrane. Thermal variations may also impact the stability of the end-product [50]. Through this approach, the complicated process of transferring the graphene growth on copper or nickel substrates cannot be prevented. In such cases, high temperatures (exceeding 1200 °C) or very high costs are mandatory and usually deteriorate graphene’s properties. Liu et al. suggested a new technique for the fabrication of graphene on different substrates (silicon or aluminum oxides etc.). This is an effective approach for the synthesis of large surface membranes [51].

The reduction of GO is a different option for the fabrication of graphene sheets. GO is created according to the Brodie, Staudenmaier and Hummers’ methods [52]. Hummers and Offeman [21] designed the Hummers’ synthesis technique with the goal of being both efficient and safe, in an effort to eliminate the creation of acid fog during the oxidation of graphite, since oxidation is required, with the help of chemical reagents, including sulphuric acid and sodium nitrate, and the product is then mechanically exfoliated in water. Graphene oxide (GO) was produced on a lab scale by Sohail et al. [53], through a modified version of Hummers’ approach. Yu et al. [54], made additional modification on the Hummers’ method, by replacing the potassium permanganate with potassium ferrate, while the sulfuric acid was regulated to lower the quantity of the reactant consumed and increase the concentration of graphite. This method is efficiently used for the synthesis of graphene to be used in supercapacitor-electrodes [45]

Hummer expanded on Brodie’s and Staudenmaier’s approaches, by establishing an oxidation phase for the production of GO [55] by combining sodium nitrate and sulphuric acid complementing the above with potassium permanganate. The end-product was more oxidized than the outcome of the Staudenmaier’s method; however, a pre-treatment was essential for the improvement of the oxidation with Hummer’s approach, as suggested by Kovtyukhova [56]. The graphite was later meticulously washed, filtered, re-washed with deionized water (DI) water and air dried, which resulted in a better-quality GO with very little non-oxidized quantities. Various modifications have been applied on the above techniques in the meantime. Staudenmaier expanded on Brodie’s results by lowering the pH level of the graphite and nitric acid mix, while carefully integrating the potassium chlorate solution into it. This improved the eminence of the resulting GO and minimized the duration of the synthesis phase. Nevertheless, it has famously long total duration, and the use of potassium chlorate produced chlorine dioxide gas, which is considered dangerous. The same acid mix was also used for the synthesis of carbon nanotubes [57] and fullerenes [58]. Consequently, this approach did not become popular. The modified Hummers’ method is the most effective option for the synthesis of graphene oxide [45]

Via reduction, the GO partially reverts to its initial state, but with improved properties, such as better electrical conductivity [59,60,61]. There are many reduction approaches, such as chemical reduction, which prepares GO and rGO high quantities, through an efficient and simple process [62], involving the immersion of GO a variety of chemical reducing agents for a specified duration and range of temperatures [60]. Thermal reduction is also effective in manufacturing high-quality rGO powder, by reducing GO at higher temperature levels (exceeding 1000 °C). This is an effective reduction mechanism; however, it cannot maintain the film form of graphene oxide [63,64,65]. UV light reduction exposes GO to UV light. Powdered GO is dissolved in a solvent because it can absorb more UV light in a liquid state, resulting in a high-quality rGO [66]. Ghorbani et al. [67] established a hydrothermal synthetic approach for the fabrication of reduced graphene oxide (rGO). The main appeal of this method is that it does not require the use of very toxic reducing agents that are dangerous for both humans and the environment. This technique calls for the reduction of graphene oxide in water at approximately 140 °C [45].

Liquid-based exfoliation of graphite is another promising method for manufacturing graphene at a larger scale. It refers to the exfoliation of bulk graphite into thin graphene while being in a liquid medium, excluding the chemical oxidation phase [68]. This approach has proven to be cost-efficient, effective, and incredibly flexible, suitable for mass production of defect-free graphene [69]. Despite multiple variations of the method emerging since its introduction [70] there are still some obstacles to the processing of graphene, because of its reduced solubility and graphene’s low colloidal stability in most frequently used solvents [71]. This also applies to graphene-based compounds, severely reducing its performance. Functionalization was suggested as a potential answer to this issue [72].

### 3.3. Challenges at Synthesis of Graphene-Based Membranes

Despite the fact that bottom-up approaches are able to produce single-layer, flawless graphene, they are not currently applicable for large-scale synthesis of graphene and graphene-based materials. As a result, and taking into account the growing demand for the mass production of graphene, top-down solutions have garnered the spotlight. Such methods traditionally require the repeated oxidation and reduction of graphite, which is an inexpensive resource [31]. Additionally, they have become more popular owing to the progress achieved in the area of direct exfoliation of graphite while using different types of solvents without any reducing or stabilizing agents. Graphite-based top-down approaches are thus more financially appropriate for large-scale applications.

Nevertheless, there are still some gaps in our knowledge regarding cost-effective top-down synthesis on a large scale, without harming the environment in the process of producing defect-free graphene. The majority of the known synthetic approaches cannot consistently regulate properties such as the size, shape, edge and layer number of the produced graphene, because of the randomness of the exfoliation, growth and assembly steps. In spite of the significant progress in the synthesis of graphene-based inorganic nanomaterials, there are still certain barriers to their large-scale application. For instance, advanced uses of graphene-based metal and metal oxide nanomaterials call for a comprehensive analysis, before understanding the interactions between the surface of graphene and the nanomaterials, since they directly affect the attributes of these resulting composites. This would undoubtedly improve the potential of the produced nanomaterials in various areas, such as biosensors and drug delivery. Multiple techniques have been employed for the synthesis of homogeneously dispersed nanomaterials, using diverse reduction and functionalization approaches. It should be noted however that there are numerous reduction and surfacing materials with adverse impact on their possible uses. It is thus crucial to examine how biocompatible and/or toxic these reductants and surfactants are, to ensure the safety of the produced materials for use in biomedical applications [31]. More specifically, the strengths and limitations of the examined approaches are summarized in Table 1 as follows.

CVD and epitaxial growth are not appropriate for the production of the graphene that is required for the resulting graphene-based nanomaterials, since they typically require large quantities of graphene sheets with a modified surface architecture. Furthermore, mechanical exfoliation via Scotch-tape is a painstaking process and does not usually result in adequate quality graphene monolayers, whereas epitaxial growth requires high-vacuum and a generally high-priced production system. The production of graphene monolayers with large surface areas is now feasible owing to the quite recent advances in chemical vapor deposition techniques; however, the growth of graphene monolayers remains a challenge and appropriate techniques have not yet been fully implemented [31].

Regardless, graphene is an innovative two-dimensional building block for the growth and assembly of metal/metal oxide nanoparticles, and the integration and collaborative effects with graphene significantly improves the performance of the resulting nanocomposite. Graphene-based metal and metal oxide composites with specific or mixed structures have thus great potential for multiple large-scale applications, and they are commercially viable. The regulated production of these nanocomposites, with specified attributes, stops the restacking of graphene nanosheets, but also offers great templates for the creation of three-dimensional porous networks with improved electrical and electronic properties. Additionally, enhancement of the produced quantity will result in the synthesis of nanocompounds, which can be calibrated for numerous possible applications. A multidisciplinary approach should thus be used to establish better protocols for the large-scale synthesis of graphene, and to achieve a higher technological maturity level [31].

Guidelines are required for the creation of graphene-based membranes that offer the optimal transport of water, while still being able to be reused in separation processes. This requires the minimization of the energy barriers upon entering the membrane. Rejection capacity may also be adjusted in a similar manner by ensuring that the inter-sheet spacing only allows a single monolayer of water. To summarize, through the optimization of the process at a molecular level, the viability of graphene oxide as a suitable material for separations (e.g., desalination, alcohol dehydration, etc.) can be maximized [85].

### 3.4. Challenges and Specified Requirements to Achieve Graphene-Based Membranes at a Large Scale

Despite the fact that bottom-up approaches are able to produce single-layer, flawless graphene, they are not currently applicable for mass production of graphene and graphene-based materials. As a result, and taking into account the growing demand for the mass production of graphene, top-down solutions have garnered the spotlight. Such methods traditionally require the repeated oxidation and reduction of graphite, which is an inexpensive resource [31].

Additionally, top-down techniques have become more popular owing to the progress achieved in the area of direct exfoliation of graphite while using different types of solvents without any reducing or stabilizing agents. Graphite-based top-down approaches are thus more financially appropriate for large-scale applications. Nevertheless, there are still some gaps in our knowledge regarding cost-effective top-down synthesis on a large scale, without harming the environment in the process of producing defect-free graphene. The majority of the known synthetic approaches cannot consistently regulate properties such as the size, shape and layer number of the produced graphene, because of the randomness of the exfoliation, growth and assembly steps. In spite of the significant progress in the synthesis of graphene-based inorganic nanomaterials, there are still certain barriers to their large-scale application. For example, advanced uses of graphene-based metal and metal oxide nanomaterials call for a comprehensive analysis, before understanding the interactions between the surface of graphene and the nanomaterials, since they directly affect the attributes of these resulting composites. This would undoubtedly improve the potential of the produced nanomaterials in various areas, such as biosensors and drug delivery. Multiple techniques have been employed for the synthesis of homogeneously dispersed nanomaterials, using diverse reduction and functionalization approaches. It should be noted however that there are numerous reduction and surfacing materials with adverse impact on their possible uses. It is thus crucial to examine how biocompatible and/or toxic these reductants and surfactants are, to ensure the safety of the produced materials for use in biomedical applications [31]

Therefore, the graphene-based nanocomposite synthesis primarily revolves around the oxidation and exfoliation of graphite oxide, which is then chemically reduced to create highly reduced graphene oxide or chemically modified graphene [31].

## 4. Green Methods of the Preparation of Graphene-Based Materials

The green reduction of graphene oxide (GO) requires the use of biocompatible components under physiological conditions of temperature and pressure [86]. Most of the substances participating in the reduction and functionalization of GO exhibit high toxicity in nature, and are considered dangerous and harmful to both human health and the environment [86]. Furthermore, the presence of even the smallest quantities of agents such as sodium borohydride, formaldehyde and hydrazine on the surface of highly reduced graphene oxide could significantly affect many of its properties and has an adverse impact on its biological applications [86,87]. Plant extracts have been gaining the spotlight as reducing agents for the environmentally-friendly synthesis (green synthesis) of metallic nanoparticles using different biological materials, owing to their low cost and the fact they are considered easy to handle [86,87].

A previous work by Mujeeb Khan et al. in 2015 described an easy and eco-friendly approach for the bio-reduction of GO using *Salvadora persica* L. roots (miswak) extract. Multiple results have verified that the biomolecules in the roots also functionalize the surface of SP-highly reduced graphene oxide, serving as a capping ligand, stabilizing it in various solvents. This method could offer a better substitute for the large-scale manufacturing of graphene and graphene-based materials for diverse applications such as electronics, nanomedicine, and bionic materials [86].

Abdulhadi H. Al-Marri et al. produced similar results: a green, single-step method for the creation of graphene/silver nanocomposites through the concurrent reduction of both graphene oxide and silver ions, using a plant extract, which functionalizes the surfaces of highly reduced graphene oxide, linking it to the silver nanoparticles. Higher amounts of silver improved the density of silver nanoparticles linked to the highly reduced graphene oxide [86].

## 5. Graphene-Based Membrane Categories Based on Microstructure

The main categories, based on their structure are: (1) porous layers, (2) laminates and (3) compounds that shall be further explored as follows:

Porous layers. By limiting the number of defects introduced via physicochemical approaches, uniform nanosized pores can be created on the graphene membrane surface, achieving outstanding selectivity in separation of gases, water desalination, and other applications. Moreover, ultra-thin, porous graphene membranes exhibit much higher permeability than other compounds. The size of the pores is a pivotal factor for use in desalination projects [27].

**Figure 2:**
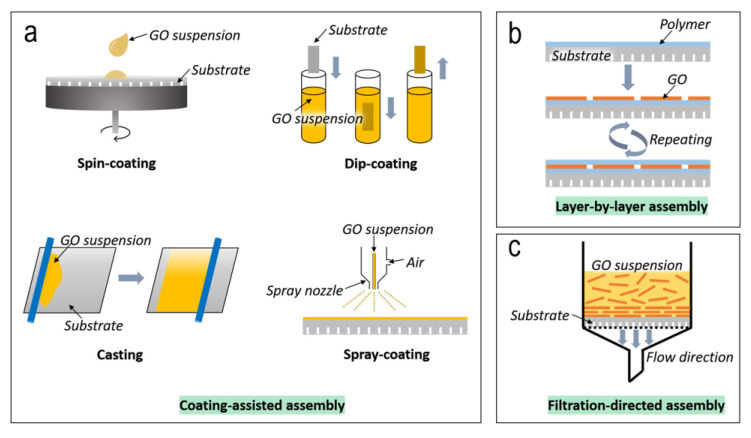
Commonly used manufacturing approaches of membranes based on graphene: (**a**) coating-assisted, (**b**) LbL and (**c**) filtration-based assembly. Reprinted with permission from Ref. [88].

Their production methods are related to specific types of manufacturing approaches. For example, coating techniques, such as spin-, spray- or dip-coating and membrane casting are widely used for the preparation of membrane layers on the substrates (Figure 2a). Due to the two-dimensional GO structure, nanosheet alignment occurs during the coating stage. The Layer-by-Layer (LbL) approach (Figure 2b) is also appropriate for the stacking of GO sheets [27]. During this process, the assembly is conducted by using deprotonated carboxyl groups of GO with other cationic electrolytes. Filtration-directed assembly is another specialized membrane synthesis technique that can be used for both GO and other 2D materials (Figure 2c) [88].

Assembled graphene laminates. Laminated membranes based on graphene use GO nanosheets as basic stackable components. There are lot of research works on the subject of the optimal production of laminated membranes with minimum defects and enhanced efficiency. Kim et al. [89] used a GO membrane with several-layer nanosheets, where the membrane permeability was impacted by various coating rounds, suggesting that the structure of the laminates depends on the careful regulation of the synthesis process. Chi et al. [90] used the same approach but under high temperature levels to speed up the evaporation of the solvent while the mixture is spun. The fabricated membrane is extremely thin and ordered. External forces [91] used during the spin-coating phase may also facilitate the nanosheet alignment.

Comparable behavior can also be achieved through a casting process. Akbari et al. [92] stated that GO laminates can be fabricated through blade-casting, which can be scaled to produce GO membranes with a surface area larger than 100 cm^2^. Tsou et al. [93] examined the impact of various filtration assembly methods on the structure and effectiveness of laminated GO membrane, concluding that the pressure-aided approach was preferable to the typical vacuum filtration when building a dense and ordered structure. A membrane was also created via simple evaporation for reference, which evidently performed worse and is not considered suitable. The GO membrane produced through pressure-assisted filtration however, exhibited great results, reaching a flux of approximately 4300 g/m^2^·h.

Graphene-based composite. GO-modified polymeric membranes have demonstrated enhanced permeability, selectivity, and anti-microbial outcomes [94]. Two main approaches have been designed to adapt the membranes, by either directly incorporating them into polymeric casting solutions during the synthesis [95,96,97], or by functionalizing the polymeric membranes via surface modification of GO nanosheets [98].

The first method has been tested by various scientists, including Lee et al. [99], who manufactured polysulfone (PSF) membrane bioreactors. A performance review indicated that the bioreactors are great anti-foulants and demonstrated a quintuple improvement in the period of time before chemical cleaning of the membrane was required. Zinadini et al. created a novel polyethersulfone (PES) composite membrane with embedded GO nanosheets [100], where the resulting membranes possessed bigger pores and were more hydrophilic as opposed to the pristine material, and demonstrated enhanced water permeability and anti-fouling abilities. Similarly, Ding et al. [101] fabricated solvent resisting membranes. Initially, a dopamine modification was applied to a polyacrylonitrile (PAN) substrate to enhance the interface bonds. It was observed that the graphene oxide sheets aligned in parallel to the polyethyleneimine (PEI) matrix, which allowed for the selective transfer of small-sized molecules, while rejecting larger ones. A novel type of thin film nano-membrane was recently developed by Lai et al. [102], who combined various amounts of GO into a PSF substrate. The resulting membrane showed improved properties, as opposed to the unmodified membrane. More specifically, 0.3 wt% GO was combined with a thin film nanocomposite (TFN) membrane resulting in a water flux of approximately 354 L/m^2^/bar/h, and better rejection rates for sodium sulfate (95%), magnesium sulfate (91%), magnesium chloride (62%), and sodium chloride (60%). Zhang et al. [95] created a GO laminate composite membrane by dissolving GO into a polyamide block copolymer (Pebax) solution. The outcome suggested that the inclusion of the laminates into the membrane considerably enhanced the performance of the initial (Pebax) membrane without affecting its selectivity, due to a marked improvement of Young’s modulus of the fabricated membrane. In addition, the composite membrane was more flexible and was found to be suitable for the mass production of the composite membrane.

Perreault et al. [103] implemented an alternative approach to enhance the thin film composite (TFC) polyamide membrane attributes by functionalizing the GO surface. GO was strongly bonded to the surface, which enabled the GO nanosheets to present at the surface and inactivated bacteria. This approach could also considerably lower the amount of GO needed for the functionalization, thus lowering the final cost. The findings indicated that the manufactured membranes demonstrated superior antimicrobic abilities, inactivating approximately 65% of the bacteria after just one hour, thus verifying that the fabricated membranes are a viable option for the creation of innovative antimicrobial membranes.

Zhang et al. [104] developed a hierarchical membrane by assembling GO sheets on the surface of aminated polyacrylonitrile (APAN) fibers, using the fiber gaps for micro-filtration of oil and water emulsions. Study results revealed that the membrane exhibited extremely high water flux due to the GO/APAN membrane being very hydrophilic. A high rejection ratio (98%) was also noted, along with outstanding fouling resistance and stability while separating oil–water emulsions at a variety of pH levels and salt concentrations. As a result, the novel GO/APAN membrane is deemed a promising option for the treatment of wastewater with oil content, as supported by a different study by Zhang et al. [98].

## 6. Graphene-Based Membranes in Water Purification

### 6.1. Graphene-Based Membranes in Water Purification

Water purification through membranes is based on the filtration, the adsorption, the rejection, and the degradation of pollutants. The efficiency of such membranes is evaluated by checking if they are water permeable, using contaminated organic solutions and deionized water. The efficiency of the prepared membrane can be impacted by the concentration of cations, the pore size, spacing between layers, chemistry of the surface, dosage, pressure and other parameters [10].

**Figure 3:**
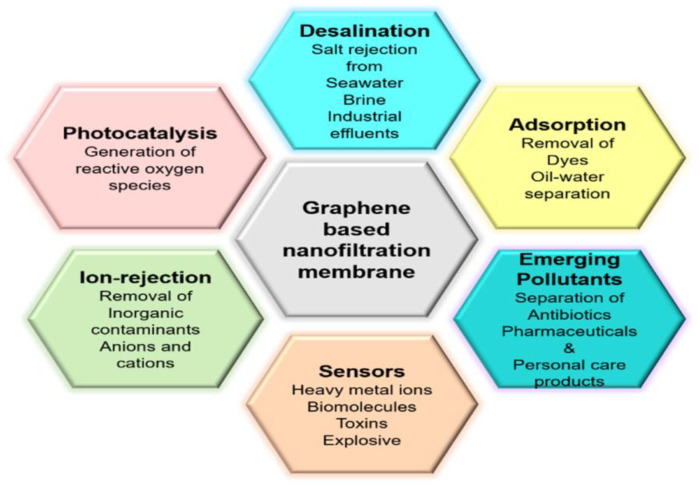
A schematic depiction of the range of water purification applications of the studied membranes. Reprinted with permission from Ref. [10].

The various applications such as desalination, rejection of ions, and pollutant removal are depicted in Figure 3. The strategies using these membranes have also been gaining researchers’ attention [10].

### 6.2. Desalination

The desalination of seawater guided by a vapor pressure gradient is a relatively new treatment approach. Despite its promising experimental results, the purification of a combination of different pollutants and keeping the temperature stable are significant setbacks that have not yet been addressed [12]. Nanofiltration is more effective in terms of water transportation [105]. A GO-incorporated polymer nanomembrane exhibits a high flux rate due to electrostatic interactions. The pore sizes permit water to go through, whereas salts or other contaminants are blocked [10].

Xu et al. [106] suggested that the surface functional groups of GO, which are negatively charged, enabled the desalination process. A GO based polymeric composite membrane exhibited improved rejection of salts and a better flux rate. When GO was oxidized at 70 °C, the highest flux was observed [10].

Romaniak et al. [107] created a graphene-based semi-permeable membrane via the in situ polymerization method for desalination. Different research discussed the use of a novel silicon oxide-GO membrane whose surface is hydrophobic, for water distillation, produced via vacuum filtration. The surface roughness improved by the mixing of nanoparticles and the hydrophobicity of the membrane was increased by grafting long alkyl chains (hexadecyl-trimethoxysilane). The desalination efficiency is consistent even at high salt concentrations [10,20].

Dong Han Seo et al. proposed a water desalination process via membrane distillation. Graphene films procured from renewable oil achieved notably better water vapor flux retention and rejection rates, and an improved antifouling capacity under a mixture of saline water that contained diverse pollutants [10,12].

### 6.3. Photocatalysis

**Figure 4:**
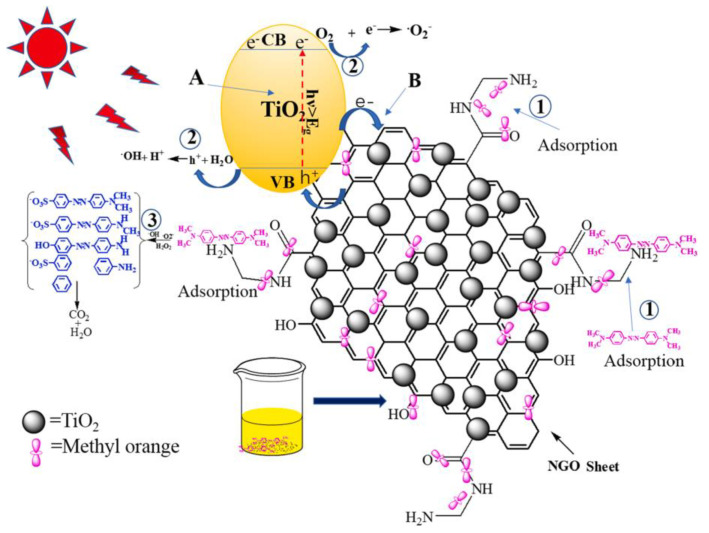
The photocatalytic process of nanostructured graphene-titanium dioxide. Reprinted with permission from Ref. [108].

Photocatalysis refers to the process of accelerating a photo-generated electron during catalysis. Recently, Chen et al. [108] examined the extraction of dyes via an aminated GO-titanium dioxide compound through the photocatalysis process. They studied the generation of photoelectrons by solar light irradiation on the prepared composite, which participated in the decay of MO dye, resulting in an improved catalytic performance, owing to the reduction in band gap energy and the increase in quantum efficiency as compared to pure titanium dioxide (Figure 4A and Figure 4B, respectively). At first, Brownian motion occurs, and many of the dye molecules are adsorbed as depicted in Figure 4. The created photoelectrons react with water and oxygen and generated hydroxyl and oxygen^-^ radicals as indicated in Figure 4.2. Furthermore, dye molecules are combined with the active groups, which fully decompose them into smaller molecules, as shown in Figure 4.3.

Yu et al. [109] described the creation of an innovative membrane, which is able to remove contaminants and has desirable antifouling properties. The prepared nanomembrane exhibited high photocatalytic activity for the reduction of chromium metal ions and oxidation of organic compounds. The findings suggested good degradation efficiency even after six rounds for mixed contaminant [10].

### 6.4. Adsorption

Materials based on graphene play a significant role for the elimination of environmental contaminants via adsorption, which relies on the bonds betwixt the pollutant and the adsorbent surfaces. Its mechanism may be based on adsorption, charge transfer or dipole interactions, electrostatic or dispersion forces, and quadrupole interactions [110,111]. The adsorption of heavy metal ions relies on physicochemical bonding interactions between the ions and the adsorbents. A recent analysis verified that graphene oxide can be successfully used to adsorb heavy metal ions [10].

Zhan et al. [112] evaluated a GO/graphitic carbon nitride/titanium dioxide foam that displayed an outstanding adsorption ability toward oil from wastewater and organic pollutants such as cationic dyes, while further displaying great antifouling properties. Dou et al. [113] verified the outstanding adsorption ability of GO-based hybrid membranes toward copper, nickel and other metal cations. Croitoru et al. [114] indicated Chitosan and GO as suitable for the elimination of heavy metal ions. The rGO membrane is found to be appropriate for environmental applications owing to its low cost and simple synthetic process [10]. Yu et al. [115] created an rGO membrane for the adsorption of copper metal cations through chemisorption. The adsorption of metal ions on the surface of the prepared membrane relies on time and PH levels [10].

### 6.5. Ion Rejection

Pollutant rejection from wastewater through membranes is based on the size of the pores and the interaction between the surface and the ions [116,117]. GO-based membranes have nanochannels that are highly permeable and display outstanding ion selectivity. Ion rejection can be regulated through nanopore functionalization. Nevertheless, the selectivity of these membranes may suffer due to swelling [10].

Attempts to prevent fouling and swelling have relied on electrokinetic effects, intercalation of ions, physical confinement of organic material molecules, pore size or channel heigh tuning, cross-linking blending with nanocarbons, and reduction of GO. The adjustment of nanochannels and nanowrinkles properties may enhance the rejection rates, because of the existence of extra water transport channels [10]. Yuan et al. [118] verified that reducing GO in the air thermally results in rejection rates up to 83%, as opposed to graphene oxide reduced in vacuum and HI. Nanowrinkles are taller (between 5 and 10 nm) than hydrated ions; thus, more nanowrinkles result in low ion rejection rates. Less graphene oxide in air widens the nanochannels, resulting in high water flux and improved ion selectivity [10,118].

Seunghyun Hong et al. demonstrated the very high charge selectivity for graphene oxide membranes. By estimating the diffusion of a wide range of ions, sizes and charges, they were able to comprehend the corresponding mechanisms and the factors affecting the ionic sieving in GO membranes, namely electrostatic repulsion and the compression of the ionic hydration shell within the membrane’s nanochannels [119].

### 6.6. Sensors

The pollution of heavy metal ions in water sources and wastewater is an issue of global concern due to their high toxicity even at very low concentrations [120]. The elimination of mercury, lead, arsenic and copper from the wastewater has garnered the attention of scientists as such pollutants are prone to end up in soil/organisms and become a part of the food chain, posing an environmental hazard [10,121].

Various approaches have been suggested to eliminate them from water; out of which, membranes based on graphene were deemed to be low cost, effortless and more effective. Wang et al. [122] demonstrated the impact of high cationic levels on GO membranes in terms of removing heavy metals. It was verified that the spacing between the membrane layers can be adjusted by regulating the cationic construction. Bandehali et al. conducted a review [123], in which functionalized GO was used to create a polyetherimide-based membrane. The prepared sample displayed notable removal of lead nitrate, copper nitrate, chromium sulphate and copper nitrate [10].

### 6.7. Emerging Pollutants

Emerging pollutants such as herbicides, pharmaceuticals and personal care products commonly detected in both potable and waste water, posing a global concern. The inability of traditional approaches of treatment is an important concern for the extraction of these emerging pollutants from wastewater. Graphene-based nanomaterials demonstrated promising potential to eliminate them from wastewater via adsorption, filtration, and photocatalysis [10].

Khalil et al. [124] produced porous graphene at comparatively low temperatures, which was deemed to serve as an effective adsorbent for the elimination of pharmaceuticals such as diclofenac. Sheng et al. [125] similarly examined an rGO-based nanocomposite membrane for in situ catalytic oxidation of sulfamethoxazole [10].

## 7. Fabrication and Performance of Graphene-Based Membranes

Various approaches were established for the manufacturing of GO membranes, such as LbL, vacuum or pressure-assisted, cross linking, intercalation, annealing and coating. Some of the recent works and their findings are summarized in Table 2.

Vacuum/Pressure-Assisted Method. Such techniques are the most frequently used and clear-cut approaches for the mass production of graphene oxide nano-, micro, and ultra-filtration membranes [126].

Cheng et al. used vacuum filtration to fabricate hybrid tungsten disulphide/GO 2D membranes to accomplish fine molecular sieving. The nanochannel size of the tungsten disulphide membrane became smaller and more regular after introducing the GO nanosheets. The hydrated hybrid membrane with 15 wt% GO showed high rejection rate of more than 90% versus dyes and ions. In addition, the nanochannels in the fabricated membrane exhibited strong rigidity under up to 0.5 MPa pressure, while the separation effectiveness slightly changed during 120 h of cross-flow filtration [127].

Sun et al. fabricated new membranes comprising of large-area, high-strength metallurgical graphene, GO, hydrazine and polyamide on porous polysulfone scaffold. The GO layer was created from GO suspensions via vacuum filtration. The effectiveness of the membranes was assessed via forward osmosis tests. The membranes showed promising results in ion blocking (>95%). The results indicated that the manufactured membranes can be useful in treating water [128].

Zhao et al. successfully prepared from GO quantum dots and sheets via a simple vacuum filtration process. The study revealed that the resulting composite displayed improved markedly improved permeability. When more quantum dots were used, the structure was not affected, while the average spacing between layers increased. Furthermore, the intercalation of the quantum dots enhanced the hydrophilicity of the membranes to a degree. More specifically, the permeability of the membranes in all cases was two to four times better without affecting their retention for direct yellow, bovine serum albumin, humic acid, and gold nanoparticles (higher than 99%). This work proves that the homo-structure of zero dimensional GO quantum dots in two-dimensional GO sheets can efficiently enhance the separation performance of GO-based nanofiltration membranes [129].

Recently Hou et al. created a GO/Methylene Blue (MB) membrane through vacuum filtration, in order to enhance the stability and rejection of dyes. The rejection rate of the GO/MB membrane reached approximately 93% for methyl orange, 99.9% for disperse black 9 and 82% for rhodamine B, whereas the pure water flux was approximately 7.7 Lm^−2^h^−1^, suggesting that the fabricated membranes have good application potential [130].

The same year Kunimatsu applied vacuum filtration to produce niobate nanosheet (NbN)- GO membranes. The impact of the weight ratio of the nanosheet versus GO on the structure and efficiency were evaluated, finding that the fabricated membranes are more structurally stable in wet conditions and were comparable with GO-rich membranes. NbN55-GO45 particularly exhibits excellent water permeability, practically six times better than the plain niobate one and two times better than a plain GO membrane, without affecting the rejection abilities of anionic dyes and salt [131].

Liu also applied vacuum filtration to manufacture a novel titanium dioxide nanorod inserted in a graphene-based membrane on a cellulose acetate mat. The nanorods extended the spacing between the graphene sheets and thus improved the separation performance of the membranes, achieving a very high flux and very high rejection rates versus dyes, exceeding 99.3 for the examined dyes [132].

Hung studied a series of graphene-modified GO membranes for nanofiltration by pressure-assisted self-assembly. The fabricated membrane exhibited high water flux, salt rejection at approximately 88%, sodium chloride at 91%, magnesium chloride at 97% and magnesium sulphate at 98% [117].

Layer by layer. Layer by layer provides a more accurate regulation of the concentration levels, the deposited film, the monolayer elasticity and the surface state. This technique, able to create nanoscale multilayer films, has thus been at the forefront of the research in various fields [133].

Arenas et al. utilized the technique to produce rGO membranes for desalination purposes and assessed the performance versus sodium and magnesium cations [134].

**Table 2:** Performance of graphene separation and decontamination membranes.

Materials	Fabrication Method	Application	Membrane Performance			Reference
			Water Permeance (LMH/bar)	Rejection (%)		
Hybrid 2D WS_2_/GO nanosheets	Vacuum filtration	Water filtration	156.3	96.30%	Methylene Blue	[127]
Semi-permeable graphene/GO membranes	Vacuum filtration	Water treatment	3.22	74.4%	Methylene Blue	[128]
				98.2%	rhodamine B	
High flux nanofiltration (NF) membranes prepared from GO quantum dots and sheets	Vacuum filtration	Nanofiltration (yellow, bovine serum albumin, humic acid, and Au NPs (>99%))	45.89	28.4	NaCl	[129]
				74.9	Na_2_SO_4_	
				98.6	Methyl orange	
GO/MB composite membrane	Vacuum filtration	Dye separation	7.67	82.60%	rhodamine B	[130]
Niobate nanosheet-GO composite	Vacuum filtration	Nanofiltration/advanced molecular separation	20	15.0%	NaCl	[131]
				60.0%	Na_2_SO_4_	
				100%	Evans blue	
Photocatalytic self-cleaning titanium dioxide nanorods inserted GO based NF membrane	Vacuum filtration	water treatment	68.1	33.0%	NaCl	[132]
				57.1%	Na_2_SO_4_	
				99.3%	Methylene Blue	
				99.3%	Methyl Orange	
GO and Graphene	Vacuum-assisted filtration		7.2	88.3%	NaCl	[117]
Laser-induced graphene/poly (vinyl alcohol)	Cross Linking	Water treatment	225	-	-	[135]
High performance hierarchically nanostructured graphene oxide/covalent organic framework (GO/COF) hybrid membranes	Intercalating imine-based COF nanoparticles.	Organic solvent nanofiltration	51−60	99%,	Methylene Blue	[136]
				99.82%	Congo Red	
oxygenated GO NF membrane	Slot-die coating	Nanofiltration	∼30	89.8%,	Methylene Red	[137]
				99.4%	Methylene Blue	
				96.8%	Briliant Blue	
				72.6%	Evans Blue	
				63.9%	rhodamine B	
Graphene oxide membrane	Mild annealing process	Nanofiltration	7.37	57.73%	Na_2_SO_4_	[138]
GO/molybdenum disulphide (MoS_2_)-PVA composite membranes	Pressure filtration	Water and landfill leachate treatment	0.592−1.416	89%	NaCl	[35]
Reduced graphene oxide membranes	Layer-by-layer	Desalination/water purification	-	27.38%	Na+	[134]
				47.44%	MG+	

Slot dye coating. This is a technique that uses GO with reduced viscosity levels, allowing for the production of thin membranes without requiring the GO solution to be pre-treated. The thin wet layer facilitates spontaneous drying, which minimizes the production time needed. Minimal waste of stock solution occurs; thus, this approach could be further examined for membrane production using various materials [2].

Kim et al. also fabricated GO membranes using a slot die coater. The membrane thickness is controllable, according to the quantity of the GO solution and morphology of the substrate. Furthermore, the coating layer and spacing are not affected, as the GO is deoxygenated (dGO). An efficiency test indicates that the manufactured membrane is effective for filtering sub-nanosized dye molecules, with a rejection percentage that reaches 99% for brilliant blue G. The fabricated membrane is stable in the case of cross-flow and high-pressure levels. The process is scalable [137].

Cross-linking methods. Cross-linking has the ability to prevent membrane swelling and dissociation, whereas the resulting membrane is also more structurally stable. Cross-linking refers to both covalent and non-covalent approaches. The covalent option improves the stability and sieving capacity of membranes based on graphene, whereas the non-covalent one assists in the dissolution of the materials [139].

Thakur et al. produced a set of cross-linked laser-induced graphene-poly (vinyl alcohol) LIG−PVA membranes. These membranes demonstrated improved selectivity and permeability and their performance was of the same levels as polymeric ultrafiltration membranes. The fabricated membranes significantly improve the strength of the LIG. Increasing the PVA levels had a positive effect on the rejection rates. The produced membranes were fouling resistant and had good flux recovery ratio [135].

Annealing process. Graphene’s properties can be impacted by either substrate interactions or the processing steps needed to manufacture contacts and devices. Annealing is used when cleaning graphene devices, which might result in doping, defect changes and strain effects [140]. The cleaning and contact annealing of graphene at increased temperature levels examines the impact of the process on graphene attributes. A detailed comprehension of the changes happening during annealing is extremely significant, and thus has been thoroughly explored by researchers [141].

A review conducted by Li et al. verified the applicability of mild annealing for creating partially reduced GO membranes and 2D laminar channels for nanofiltration. The membrane changes were methodically characterized, and results show the process not only made GO membranes more stable but also facilitated the customizable tunable salt rejection of the resulting membranes. Mild annealing rGO membrane (MRGO) accomplished better water permeance and sodium sulfate selectivity [138].

Large molecule intercalation. Due to the high surface area of GO nanosheets, the interlayer electrostatic repulsion may destabilize the GO membranes. To this end, the insertion of large molecules that may intercalate between layers can manage to address the issue and stabilize the structure. This process can also be used to tune the membrane effectiveness by adjusting spacing and enhancing molecular separation [142]. The most commonly used intercalating nanomaterials include particles such as metal or covalent organic frameworks, NH_2_-Fe_3_O_4_, and silver, as well as carbon and titanium dioxide nanotubes.

Chen et al. created a hierarchical (i.e., with nano-, micro- and meso-pores) graphene oxide/covalent organic framework (GO/COF) membrane through the intercalation of COF particles, which possess a hollow structure and nanopores at the almost 2 nm range, as well as multiple functional groups that render it extremely hydrophilic, thus offering additional fast transport nanochannels. The fabricated membrane has outstanding flux at 1 bar and excellent rejection efficiency of dyes. Furthermore, the intercalation of COF is able to considerably reduce the swelling, thus improving membrane stability when exposed to either water or strong acidic/base solutions [136].

## 8. Factors Influencing the Separation

Impact of Interlayer Spacing. The spacing between the layers of graphene is approximately 0.34 nm, whereas that of GO is about 0.8 nm [143]. If the GO interlayer spacing becomes larger, the separation efficiency deteriorates, while a smaller value allows the water molecules through but can prevent the transfer of other ions [144]. Abraham et al. examined the variations of spacing between layers in GO membranes at various environments [144]. The results indicated that the spacing grows when the relative humidity (RH) increases, reaching the upper limit of 0.98 nm at 100% relative humidity. It can be controlled by the introduction of graphene flakes amongst the GO layers, which reduces the spacing and obstructs the ion passage (97% rejection for Sodium Chloride) without impacting the passage of water significantly. Mi et al. assessed the differences in spacing in wet conditions [143]. The findings indicated that the graphene oxide that was cross-linked with ethylenediamine exhibited a controlled interlayer spacing. Ethylenediamine was selected due to its small size that was expected to result in a limited increment in spacing. In reality, it requires a few nanometers, and, in water, the spacing could be increased by up to 8 nm. GO would sustain significant changes in interlayer spacing when moving from about 0.8 nm in dry conditions to approximately 2 nm in 75% relative humidity [145]. Chen et al. demonstrated that the spacing between layers can be adjusted through various salt solutions, such as potassium and sodium chlorides [146]. The dispersion of GO in the solution has the smallest effect on the interlayer spacing when using potassium chloride and the highest when using magnesium chloride, which was measured in to be in the range of 1.2 to 1.3 nm. As a result, a wide range of spacing might be achieved by carefully choosing the cations offering the strongest interactions.

Impact of Crosslinking. Crosslinking GO layers, adapting the temperature levels and functionalization, are some of the few factors that can impact the preferred layer spacing. Hung et al. created a GO through treatment with diamine based catalysts [147]. The findings indicated that the GO/ethylenediamine (EDA) membrane had the lowest spacing difference between dry and hydrated conditions (from 0.91 to 0.93 nm) [147]. Butylenediamine (BDA) and p-phenylenediamine (PPD) diamines resulted in higher differences. The impact of crosslinking on the spacing between layers relies on the structure and attributes of the reactants. A different work by Jia et al. examined GO membranes that were created by crosslinking with various acids, such as oxalic, succinic acid and hexanedioic acids [148]. Zhang et al. manufactured GO membranes for a variety of dyes and metal removal applications [149].

Stability. The large-scale applications of graphene membranes is a daunting task, requiring the careful preparation of large area porous films, taking into account parameters such as flexibility, ability to handle easily and pore uniformity while designing the process [150]. Cleaning of the nanofiltration membrane might require the use of multiple techniques. The stability of the membrane is a critical factor under a wide range of conditions [14]. Thebo et al. examined the stability of GO membranes, using crosslinked rGO that was treated under a variety of harsh conditions [151]. Their findings indicated that the membranes were very stable in very low and very high pH levels, after 30 days of treatment. The chemical reactions might also affect the membrane stability, as discussed by Joshi et al. who suggested a model for stable rGO or GO membranes [152]. Nam et al. explored the subject of stability after using sonication at various pH levels [153]. The membranes were destroyed after half an hour of sonication in extreme conditions such as very high and very low pH levels. Crosslinking with polyethyleneimine, resulted in increased stability under all circumstances. At low pH levels, exposing the GO sheets to water makes the π-π interaction weaker; thus, the sheets are susceptible to sonication, whereas for graphene oxide-branched polyethylenimine (BPEI/GO), there are no free groups available at increased pH values; thus, the optimal stability is accomplished in alkaline settings.

## 9. Conclusions

In this work, we explicitly discuss the progress of membranes based on graphene. A large number of research works over the past few years verified that they exhibit outstanding permeability, allowing for very fast and highly-selective transfer of water and thus have very successful wastewater treatment applications through various synthesizing techniques such as the coating, layer by layer, vacuum/pressure-assisted and annealing methods. Nevertheless, there are still certain challenges to address regarding the spacing between layers and the maintenance of the structure in order to be able to exclude small-sized ions. Furthermore, avoiding membrane swelling when dissolved in wet environments is an additional issue that could hinder its usage. Important advancements have been achieved on the separation mechanisms, and various successful liquid separation applications have been highlighted. (a) Their long-term stability is still a significant issue. For large-scale projects, the testing period for their stability ranges between a few hours and a few days, which does not correspond to industrial settings. Thus, the subject needs to be further explored. (b) Secondly, the fabricated membranes must withstand chemical cleaning, since they are required to address fouling. There are still only a few membranes based on graphene that can tolerate such processes. (c) Lastly, it remains difficult to maintain the efficient separation of the membranes in liquids. The spacing of graphene sheets is affected in wet environments.

The inability of graphene-based membranes to extract a broad variety of pollutants from wastewater in just one step is an important hindrance that translates to requiring additional exploration to fix the water treatment issue. The diminishing membrane performance owing to fouling is a serious concern that needs to be appropriately addressed. In spite of the technological growth in terms of production methods, the full elimination of defects is crucial before implementations at a larger scale.

## Figures and Tables

**Table 1:** Strengths and Limitations of various methods used to synthesize graphene.

A/A	Synthesis Process	Method	Strengths	Limitations	Economic Considerations
1	Chemical vapor deposition (CVD)	Bottom up	Allows for large-scale synthesis of single-crystal graphene [73]	Emits toxic gaseous by-products during reaction [74]	Requires highly expensive equipment [74]
2	Epitaxial growth	Bottom up	High-quality graphene with excellent properties, [75]	Energy intensive [76] Difficult to control at elevated temperatures [76]	Expensive process [77] High cost of the substrates [78]
3	Wet chemical synthesis (for ex the Hummers’ method)	Bottom up	Transparent conductive film, that can be used to synthesize graphene [79]	The Hummers’ method produces nitrogen dioxide, dinitrogen tetroxide causes heavy metal pollution. Additionally, the products contained sodium and nitrate anions, which were not easy to remove [80]	The process is expensive in terms of time, energy, and waste treatment [81]
4	Mechanical Exfoliation	Top down	Time saving method [82]	It is uncontrollable and not scalable [78]	Inexpensive method [82]
5	Liquid exfoliation	Top Down	Scalable method and inexpensive [83]	Yield that is not sufficient for industrial applications at macroscopic scale [84] Other disadvantages include, toxic and the reduction of the size of the nanosheets [84]	The required solvents are expensive [84]

## Data Availability

Not applicable.
